# Supplementary material for: Templated growth of oriented layered hybrid perovskites on 3D-like perovskites
Source: Nat Commun. 2020 Jan 29;11:582. doi: 10.1038/s41467-019-13856-1 (PMC6989653; doi:10.1038/s41467-019-13856-1)
Supplement: Supplementary file 1 — Supplementary Information [file 41467_2019_13856_MOESM1_ESM.pdf]

Supplementary Information

Templated Growth of Oriented Layered Hybrid Perovskites on 3D-like Perovskite

Wang *et al.*

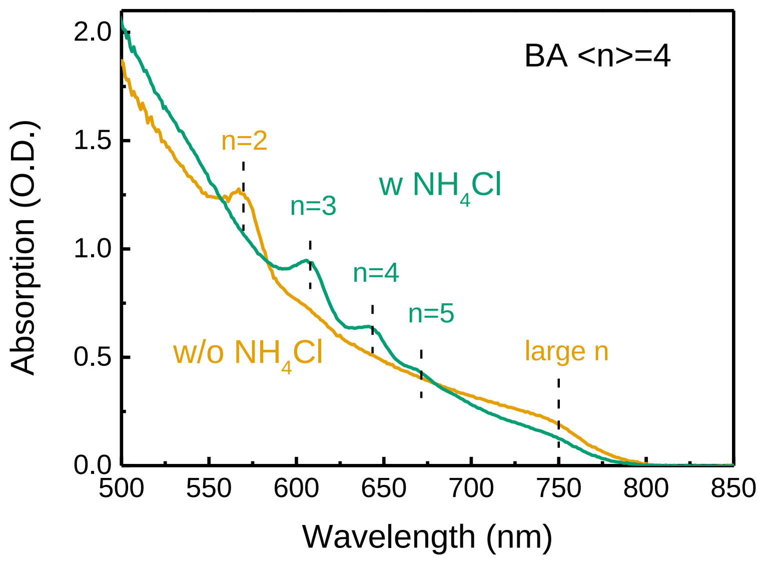

Supplementary Figure 1 | Absorption spectrum of Ruddlesden-Popper (RP) perovskite films based on butylamine (BA) cation with <n>=4 fabricated from precursor solution with or without 0.5 molar ratio of NH_4Cl additives. Though the precursor solution was composed as $BA_2MA_3Pb_4I1_{13}$ (n=4), the result RP perovskite film contained RP perovskites with different layer number n [1]. Thus, we define average layer number <n> by the component in precursor solution.

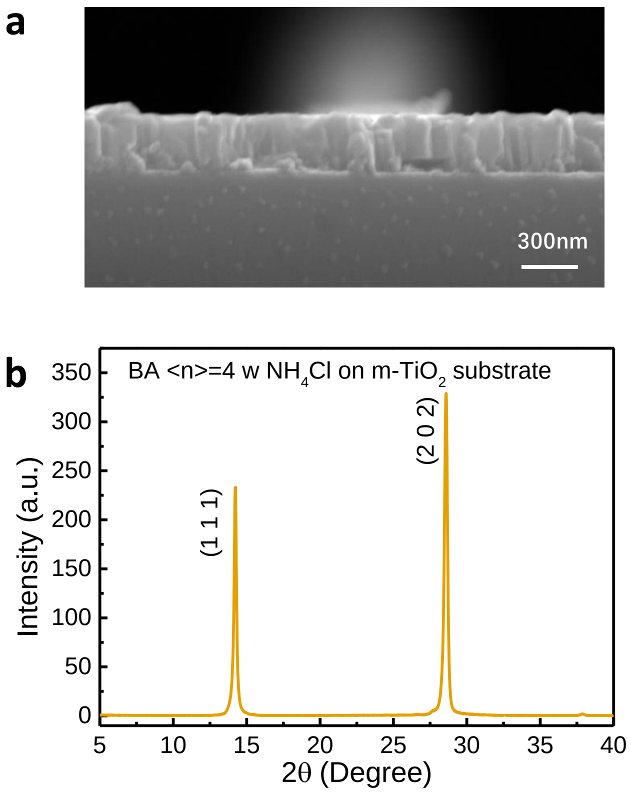

Supplementary Figure 2 | Out of plane (OP) orientation achieved for BA-based RP perovskite on meso-porous TiO_2 substrate. (a) Cross-sectional SEM and (b) XRD of RP perovskite film based on BA cation with <n>=4 and NH_4Cl as additive. OP orientation of RP perovskite on mesoporous TiO_2 substrate was confirmed by cross section SEM where vertical grain boundaries were observed and by XRD patterns where only (1 1 1) and (2 0 2) were present.

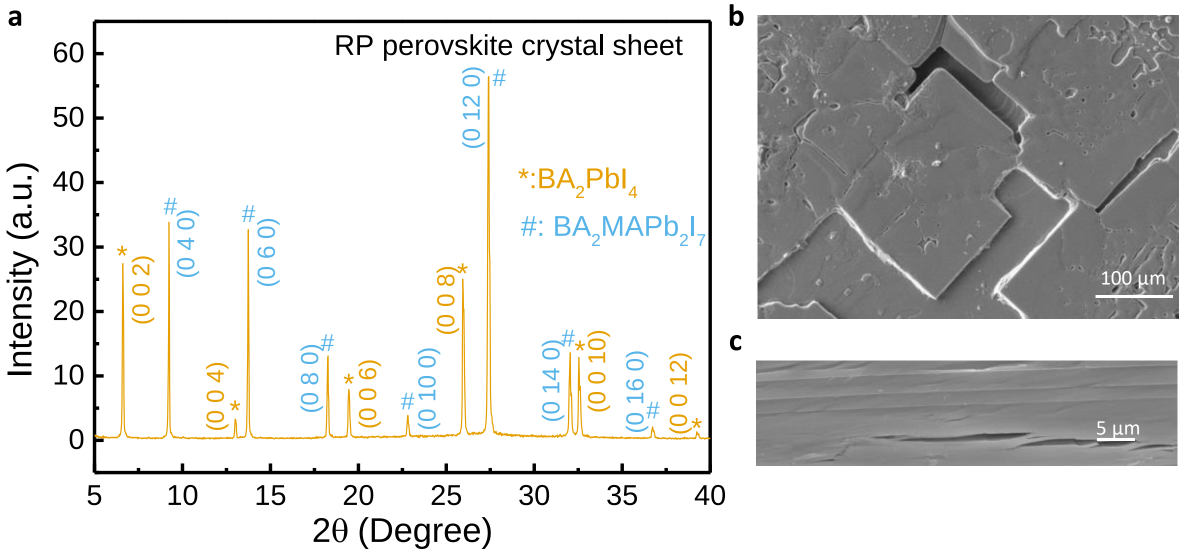

Supplementary Figure 3 | IP oriented RP perovskite grown at liquid-air interface. (a) XRD, (b) topography SEM image and (c) cross-sectional SEM image of thin RP perovskite crystal sheet grown at the liquid/air interface of oversaturated aqueous solution of BA-based RP perovskite precursor (<n>=2). The RP perovskite thin crystal sheet grown at the liquid/air interface of oversaturated aqueous RP perovskite precursor solution (<n>=2) by the slow cooling method was characterized by XRD and SEM (Supplementary Figure 3). The XRD peaks of (0 k 0) planes of BA <n>=2 and (0 0 l) planes for BA <n>=1 indicated the IP orientation of RP perovskites.[2] The layered structure with IP orientation can also be recognized from the topography (Supplementary Figure 3b) and cross-sectional SEM images (Supplementary Figure 3c), which agrees with the XRD result.

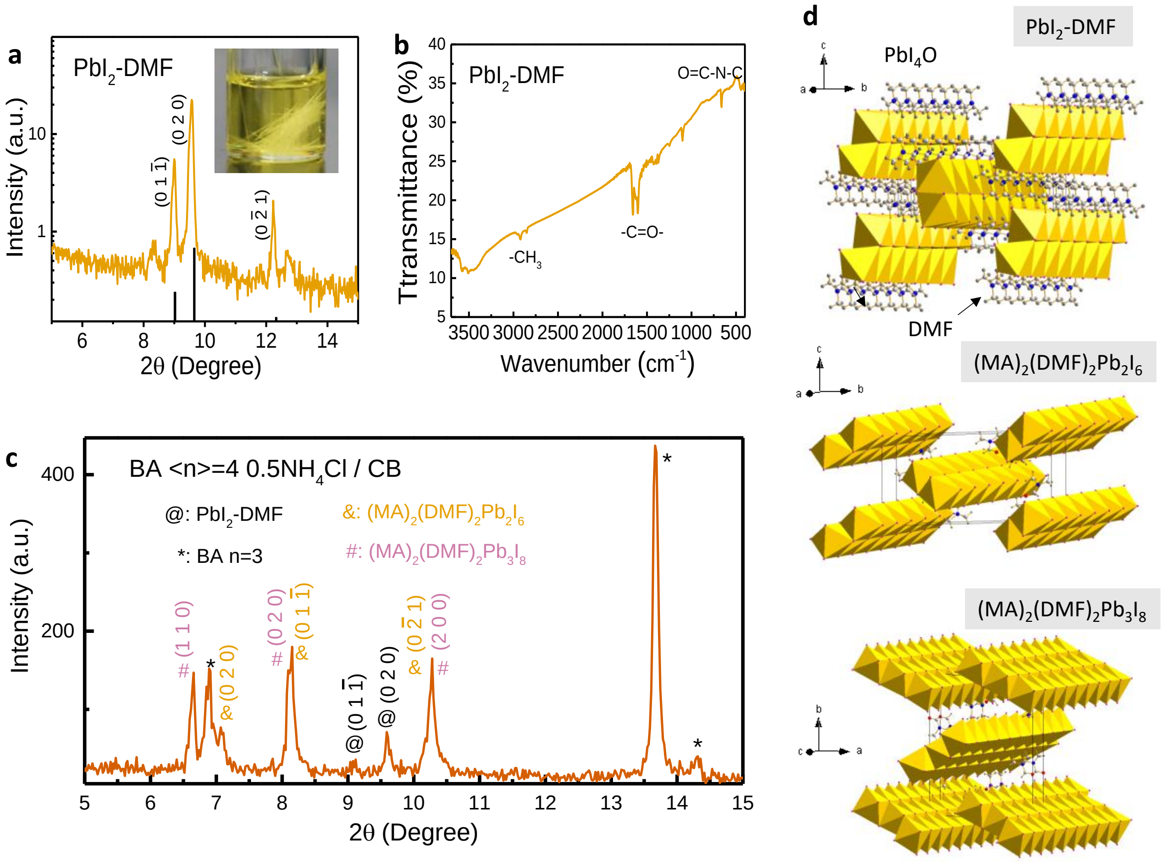

Supplementary Figure 4 | PbI_2-DMF-contained solvated phases (PDS). (a) XRD spectrum of pure PbI_2-DMF fibers. The predicted position of (0 1 $\bar{1}$), (0 2 0) and (0 $\bar{2}$ 1) peaks of PbI_2-DMF also shown (black lines) and inset showed photo of PbI_2-DMF formed in PbI_2:DMF solution at RT. (b) FTIR spectroscopy of PbI_2-DMF crystal, indicating the presence of DMF molecules. (c) XRD patterns of the precipitation obtained from BA <n>=4 0.5NH_4Cl precursor solution by antisolvent method, where the precipitation of PDS include PbI_2-DMF and MAI- PbI_2-DMF (i.e. $(MA)_2(DMF)_2Pb_2I_6$ and $(MA)_2(DMF)_2Pb_3I_8$.) phases. (d) Scheme of crystal structures of PbI_2-DMF, $(MA)_2(DMF)_2Pb_2I_6$ and $(MA)_2(DMF)_2Pb_3I_8$ solvated phases.

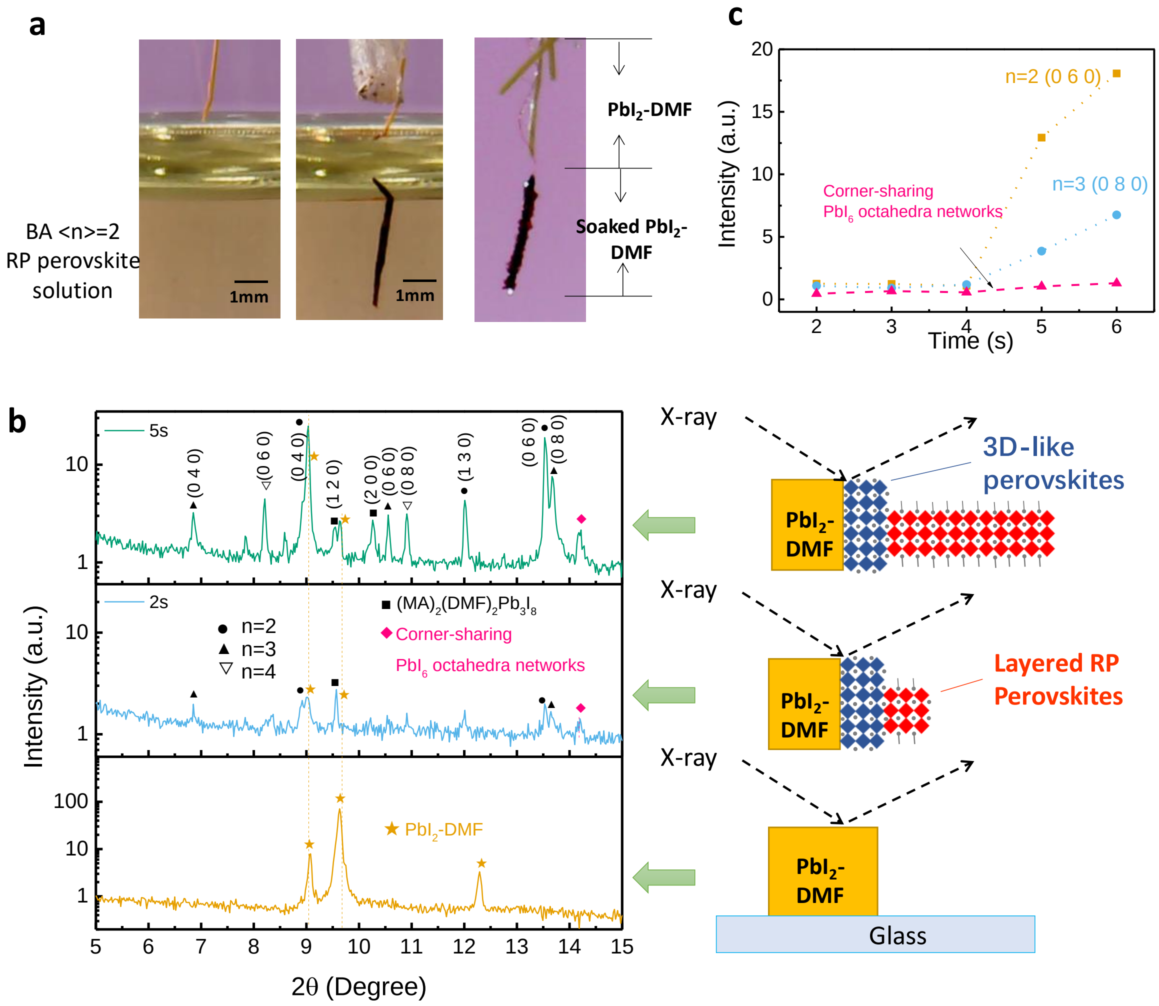

Supplementary Figure 5 | XRD characterization of PbI_2-DMF fiber before and after soaking in RP perovskite precursor solution. (a) Soaking of PbI_2-DMF phase in oversaturated BA-based RP perovskite precursor solution (<n>=2) by few seconds, which leads to the formation of black colored corner-sharing PbI_6 octahedra networks (i.e. 3D-like perovskite phase) on PbI_2-DMF fiber together with few RP perovskite grown on surface. (b) XRD spectra of PbI_2-DMF fiber before and after soaking in BA-based RP perovskite precursor solution (<n>=2) with different time. The right panel illustrate the incident X-ray and the cross-section of the PbI_2-DMF fibers. The diffraction

peak of 2θ=14.18° was assigned to corner-sharing PbI_6 octahedra networks with a lattice constant of ~6.3 Å. (c) Comparison of the peak intensity against soaking time of the peaks of 2θ=13.53° (i.e. (0 6 0) peak of RP perovskite with n=2), 2θ=13.68° (i.e. (0 8 0) peak of RP perovskite with n=3) and 2θ=14.18° (i.e. from corner-sharing PbI_6 octahedra networks), respectively.

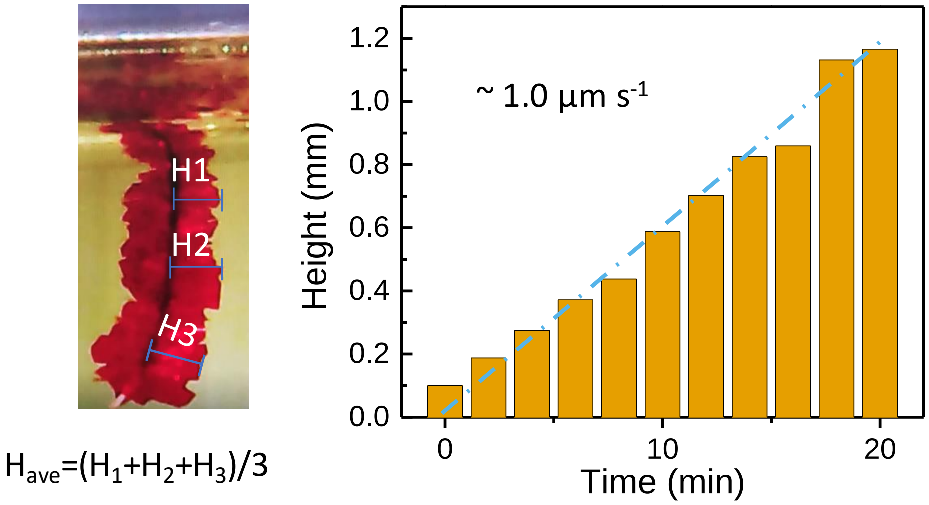

Supplementary Figure 6 | Estimated growth rate (~1.0 μm s^{-1}) of RP perovskites on PbI_2-DMF fiber. The height of the RP perovskite crystal (H_{ave}) was obtained by averaging the height of H1, H2 and H3 at the locations marked in the left photo.

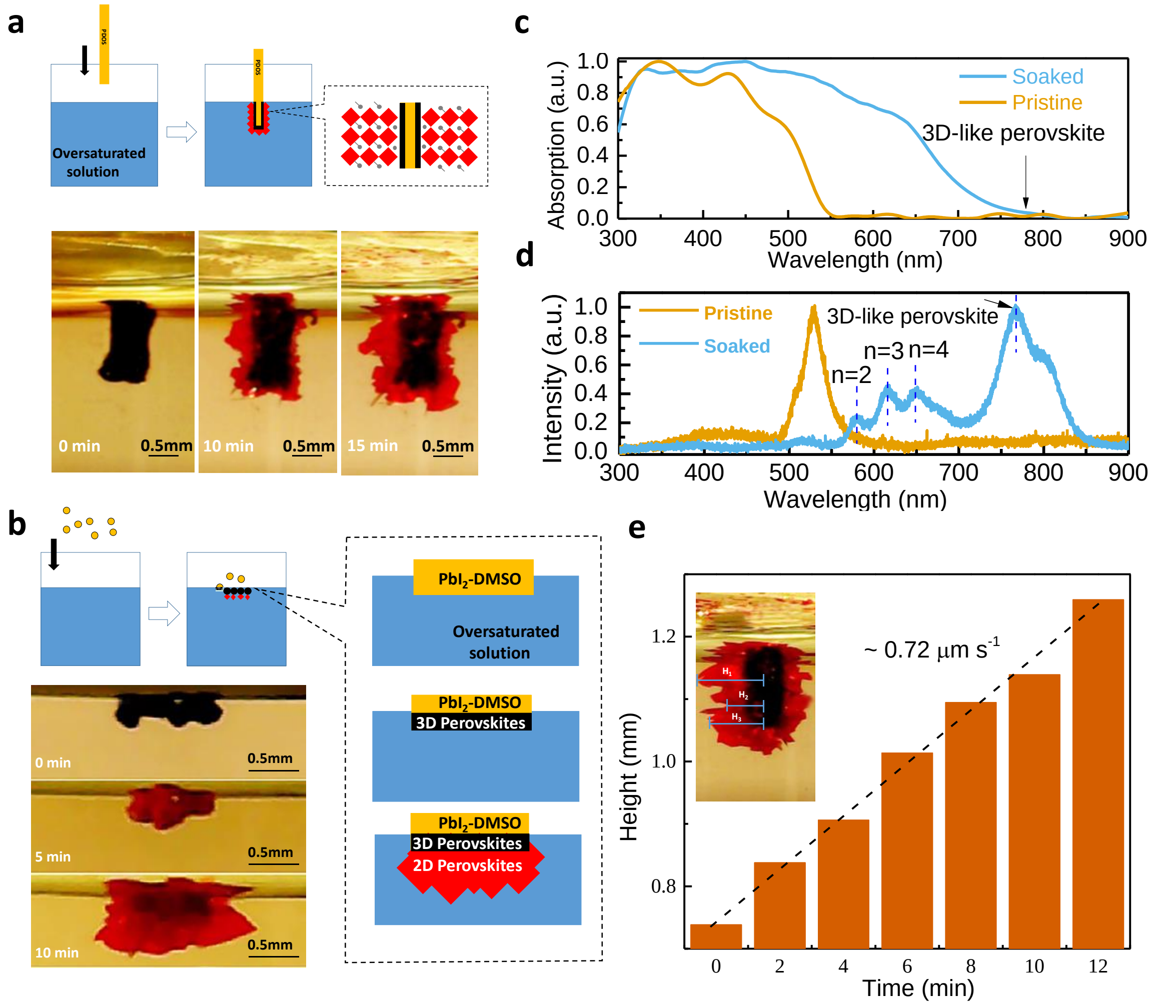

Supplementary Figure 7 | Templated growth of 2D perovskite on PbI_2-DMSO based solvated phase. Illustration and photos of the templated growth of BA-based RP crystals on (a) A PbI_2-DMSO solvated phase fiber soaked in the oversaturated precursor solution (<n>=2) and (b) PbI_2-DMSO solvated phase powders dropped on the surface of the oversaturated precursor solution. (c) Absorption and (d) photoluminescence spectra of PbI_2-DMSO solvated phase fibers before and after being soaked in oversaturated 2D perovskite. (e) Estimated growth rate (~0.72 μm s^{-1}) of 2D perovskites on PbI_2-DMSO solvated phase fiber.

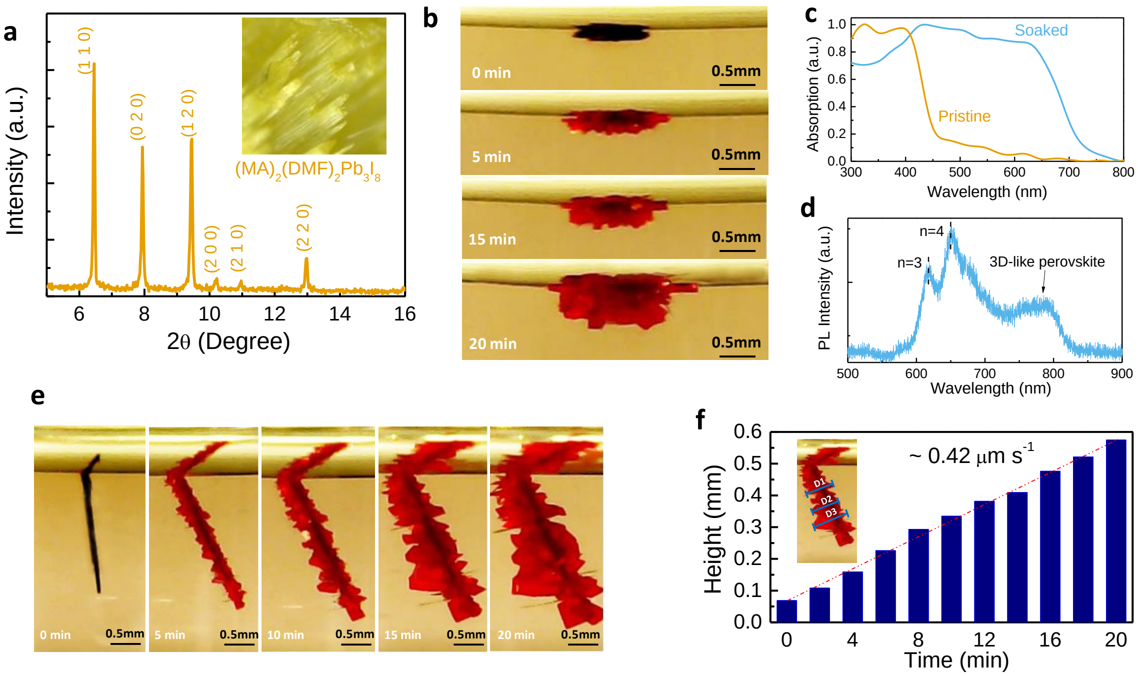

Supplementary Figure 8 | $(MA)_2(DMF)_2Pb_3I_8$ intermediate phase triggered templated growth of 2D perovskite. (a) XRD patterns of $(MA)_2(DMF)_2Pb_3I_8$ fibers with its photograph shown as inset. (b) Templated growth of 2D perovskite triggered by $(MA)_2(DMF)_2Pb_3I_8$ powders dropped on the surface of oversaturated 2D perovskite precursor solution. (c) Absorption and (d) Photoluminescence spectra of $(MA)_2(DMF)_2Pb_3I_8$ fibers after soaked in the oversaturated 2D perovskite precursor solution. (e) Inserting $(MA)_2(DMF)_2Pb_3I_8$ fibers into the oversaturated precursor solution to induce the templated growth of 2D perovskite. (f) Measured crystal size against growing time and the corresponding estimated growth speed of RP perovskites.

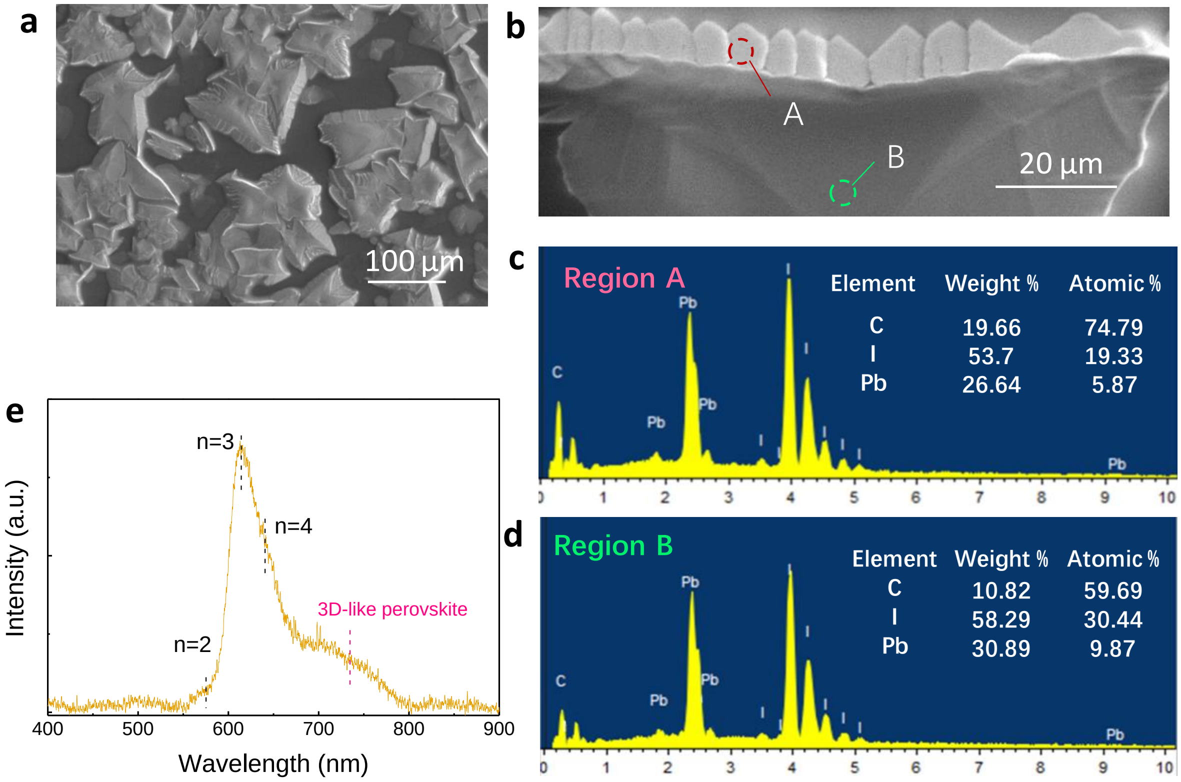

Supplementary Figure 9 | Components analysis of RP perovskite grown on 3D-like perovskite coated PDS particles. (a) SEM images of the particles with PDS in bulk and RP perovskite grown on edges, which is formed by dropping RP perovskite precursor solution into CB antisolvent. (b) Enlarged SEM image around the edge of PDS in particles, from which the nucleation and directional growth of RP perovskites can be recognized. The areas dominated by RP perovskite and PDS are noted as region A and Region B, respectively. (c, d) The presence of Pb, I and C elements in the region A (c) and region B (d) measured by energy dispersive spectrometer (EDS). (e) Photoluminescence (PL) spectra of the particles shown in Supplementary Figure S9a where the peak at 750 nm is from the 3D-like perovskite phase.

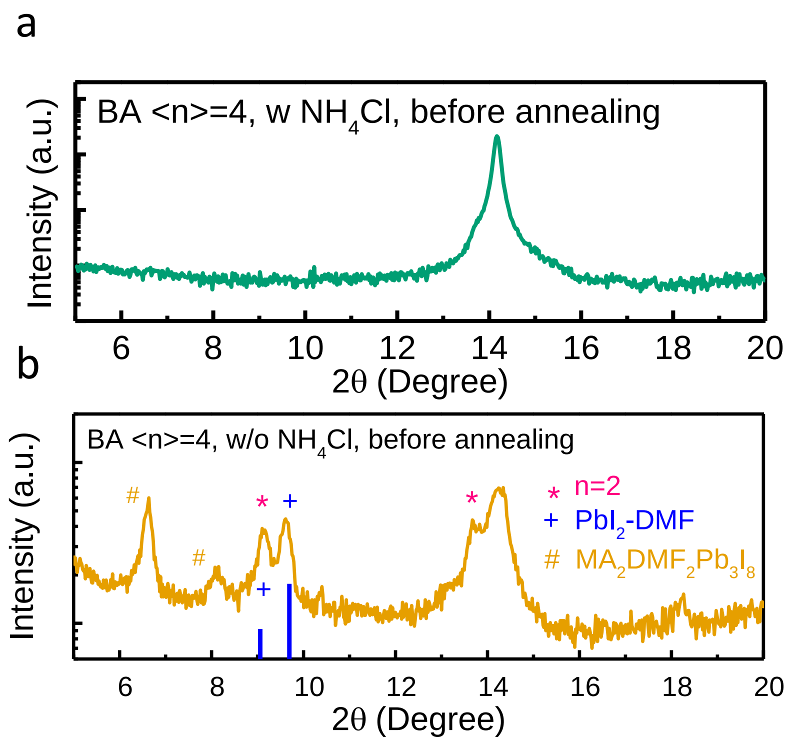

Supplementary Figure 10 | XRD patterns of unheated RP perovskite films with and without NH_4Cl additive, where the PbI_2-DMF and $(MA)_2(DMF)_2Pb_3I_8$ phases become significant in the (unheated) samples w/o NH_4Cl additive.

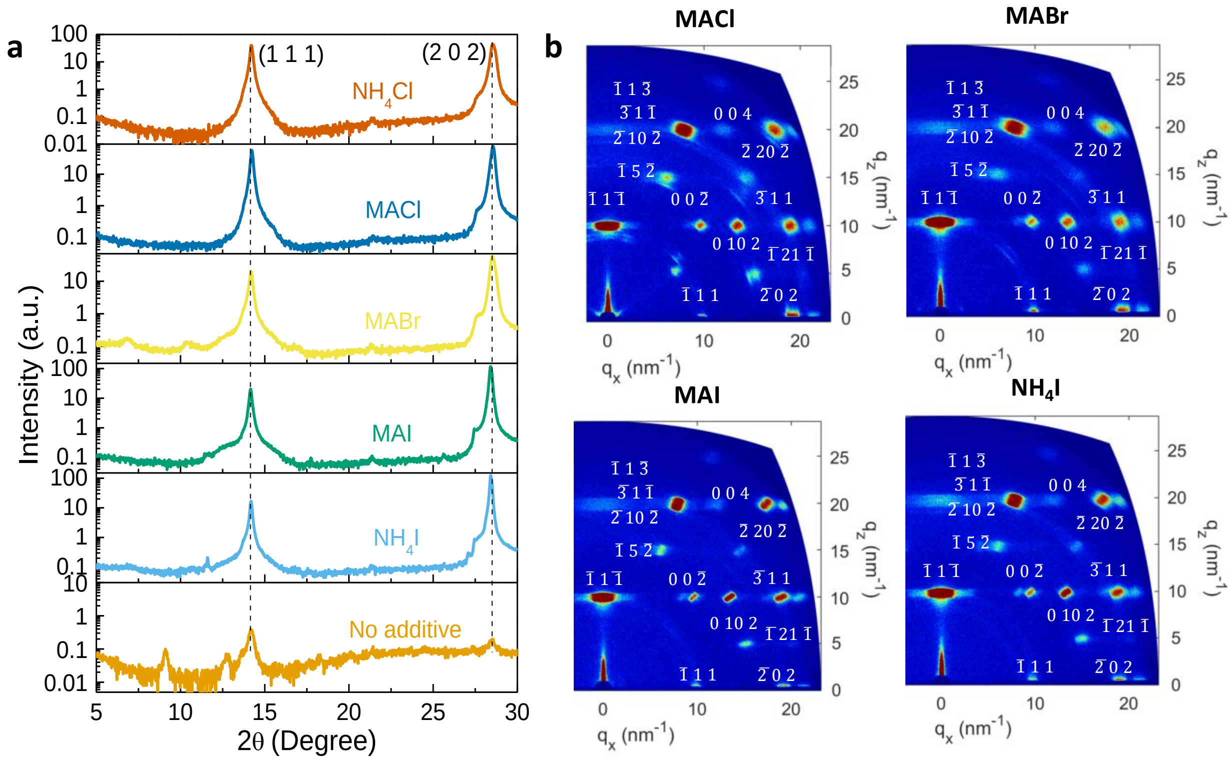

Supplementary Figure 11 | Orientation of RP perovskite films with different additives. XRD spectra of BA-based RP perovskite films (<n>=4) with MACl, MABr, MAI and NH_4I as additives. (a) XRD spectra and (b) GIWAXS patterns of BA based RP perovskite films (<n>=4) with MACl, MABr, MAI and NH_4I as additives.

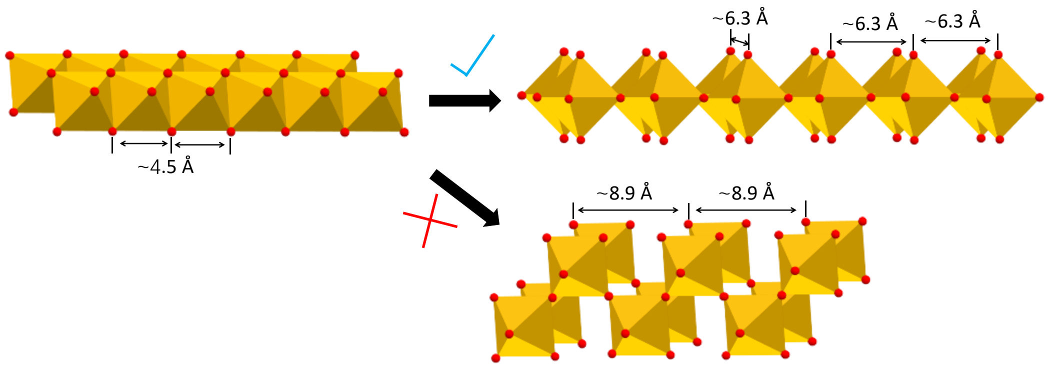

Supplementary Figure 12 | Illustration of the available corner-sharing PbI_6 octahedra chains (top) formed by rotating the edge-sharing Pb-I based octahedra chains.

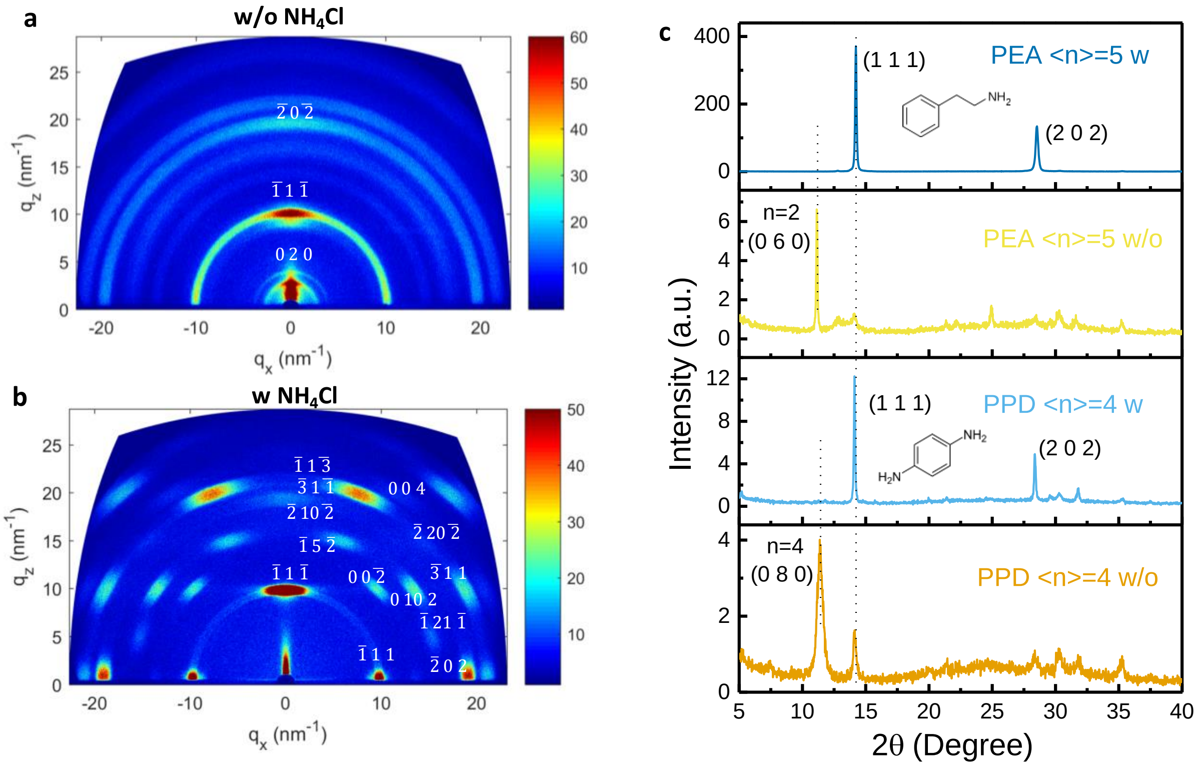

Supplementary Figure 13 | NH_4Cl additive induced OP orientation in phenylethylammonium (PEA) based RP-type and p-phenylenediamine (PPD) based Dion-Jacobson (DJ) type layered perovskites. (a,b) GIWAXS patterns of PEA based RP perovskite films (<n>=5) without (a) and with NH_4Cl as additives (b), respectively. (c) XRD spectra of PEA-based RP perovskite films and PPD based DJ perovskite films with and without NH_4Cl as additive.

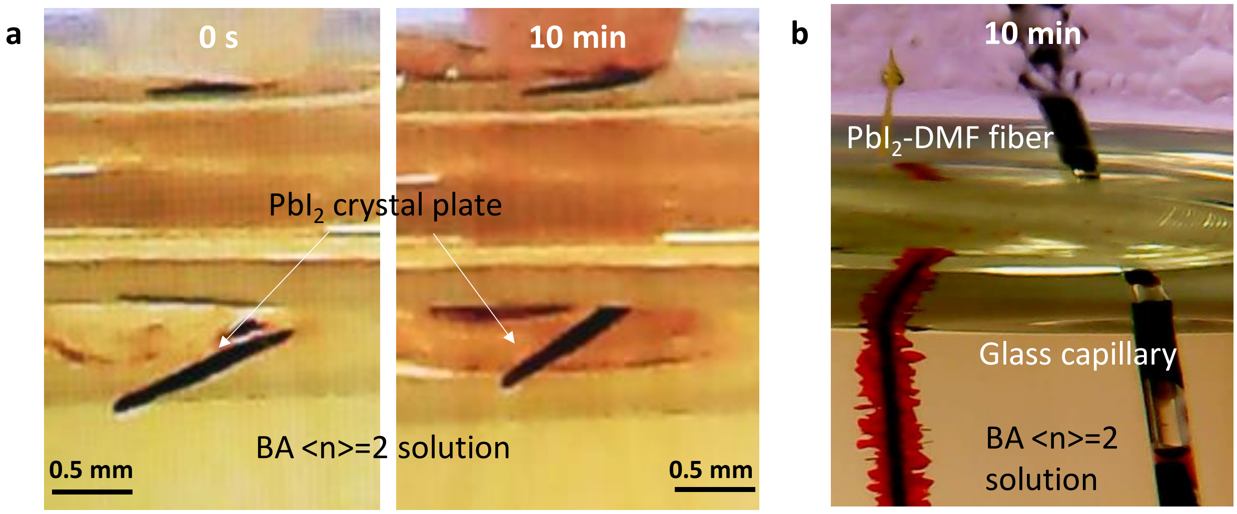

Supplementary Figure 14 | Oversaturated RP precursor solution do not trigger templated growth of RP perovskites on PbI_2 crystal. Soaking PbI_2 crystal plates (a) and glass capillary (b) into oversaturated BA-based RP perovskite precursor solution (<n>=2) for 10 mins, during which no templated growth of RP perovskites was found. For the purpose of making the glass capillary visible, black ink was sealed inside the glass capillary. The templated growth of RP perovskites from PbI_2-DMF fiber is significant (b) at the same condition. This result suggests that the low-coordinated I^- ions is necessary to trigger the nucleation and growth of RP perovskites.

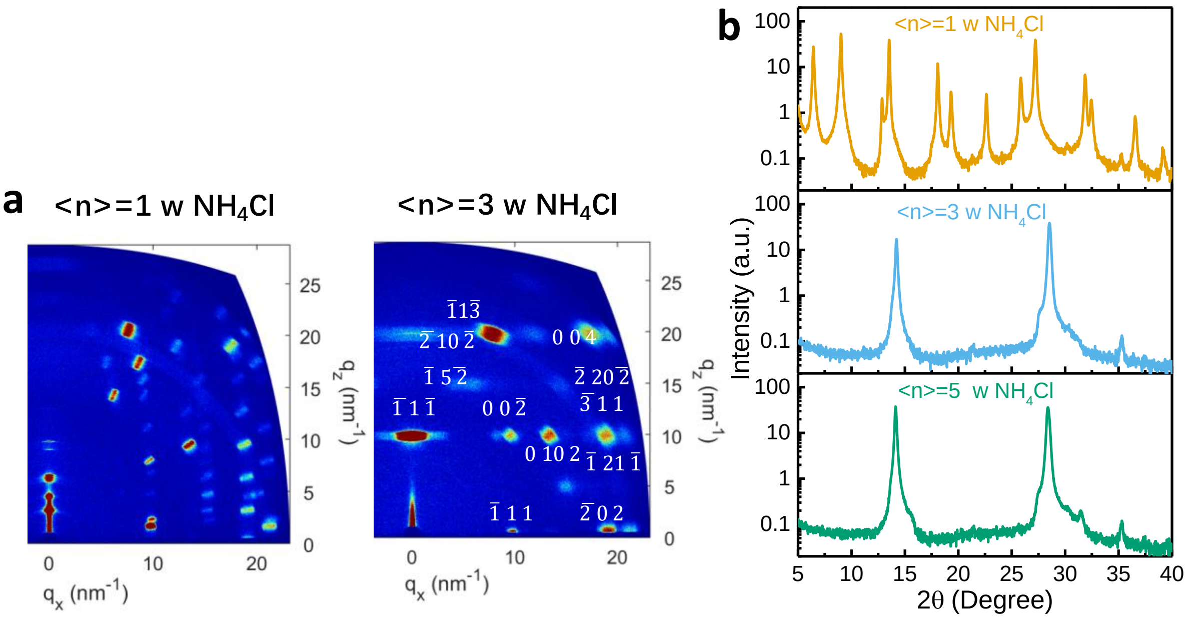

Supplementary Figure 15 | IP orientation of n=1 RP perovskite film with NH_4Cl. (a) GIWAXS patterns and (b) XRD spectra of BA-based RP perovskite films with different layer numbers obtained by spin coating in the presence of NH_4Cl additive. The strong diffraction spots at q_z<10 nm^{-1} and the XRD peaks at 2θ<10° indicates significant crystals with IP orientation in RP perovskite film with <n>=1.

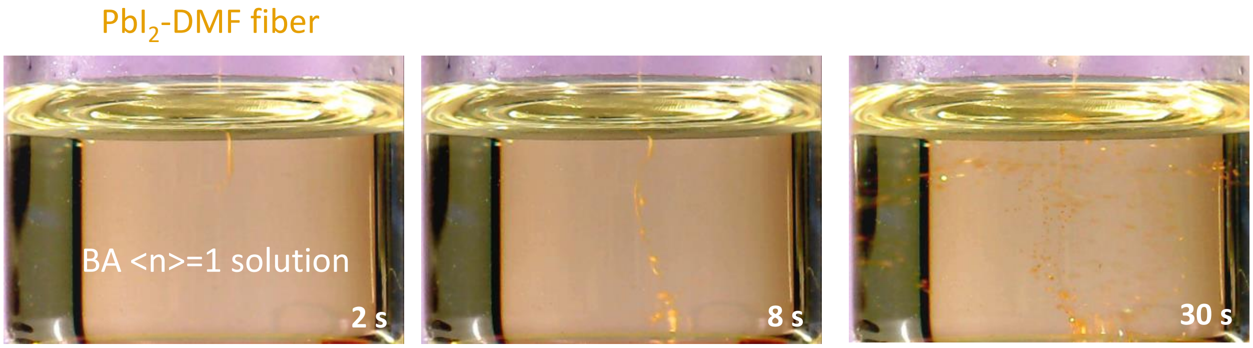

Supplementary Figure 16 | Soaking of PbI_2-DMF fiber in oversaturated BA-based RP perovskite precursor solution (<n>=1, i.e. no MA^+ ions). By tens of seconds, the formed BA_2PbI_4 crystal fragments peeled off from PbI_2-DMF and adopt random orientation in the solution. This phenomenon indicated that MA^+ ions are necessary for the templated growth of PbI_2-DMF perovskites on PbI_2-DMF.

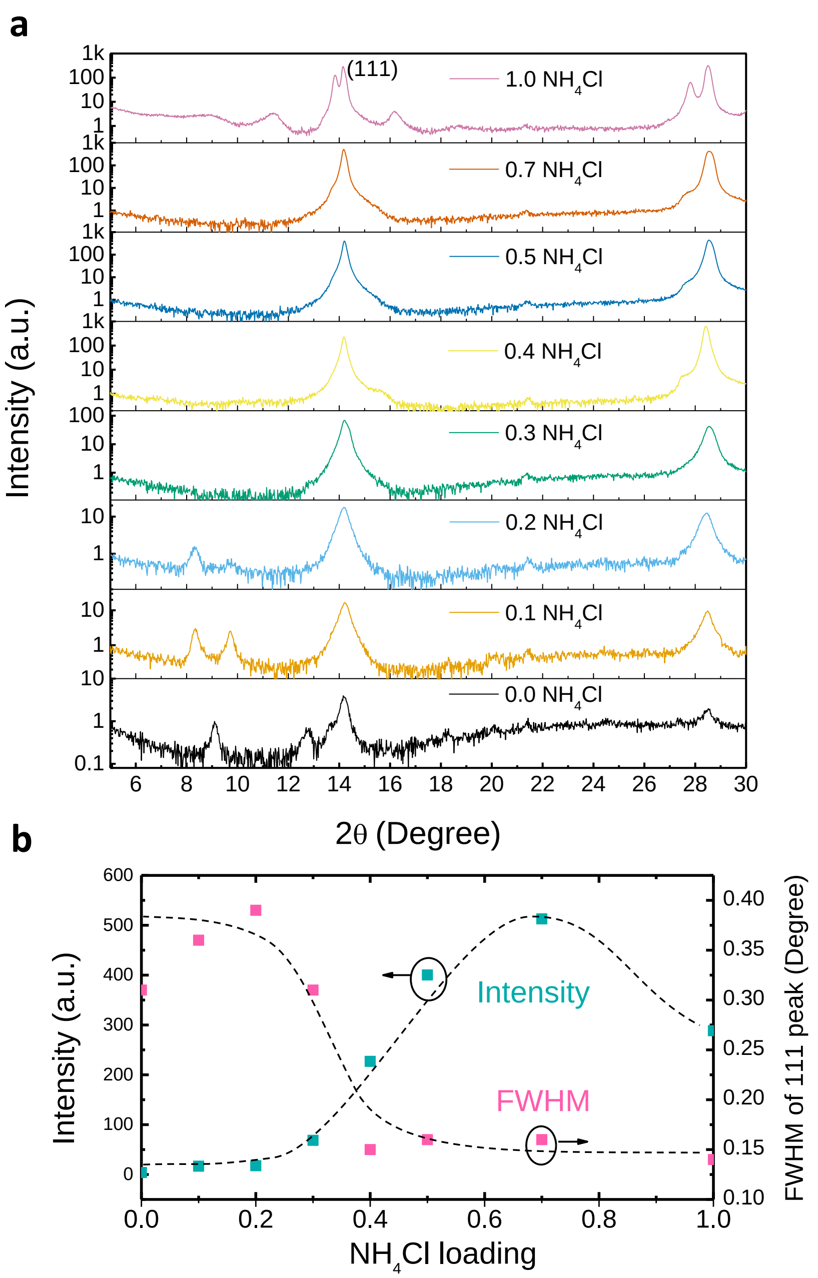

Supplementary Figure 17 | XRD characterization of <n>=4 RP perovskite films with different ratio of NH_4Cl. (a) X-ray diffraction (XRD) spectra of BA-based RP perovskite films (<n>=4) with different NH_4Cl loading (defined as the molar ratio of NH_4Cl/PbI_2). (b) Impacts of the NH_4Cl additive loading on the intensity and FWHM of the (1 1 1) diffraction peaks of BA-based RP

perovskite films (<n>=4). Supplementary Figure 17 shows the impacts of the NH_4Cl additive on the crystallinity and orientation of BA-based RP perovskite films. The point of NH_4Cl/PbI_2=0.3 is the critical additive loading for the increased crystallinity and disappear of resolvable RP perovskite crystals with in-plane (IP) orientation. The achieved FWHM of (1 1 1) peak (about 0.15°) is smaller than that of RP perovskite films from hot casting method (>0.2°) [3], suggesting a high degree of crystallinity introduced by NH_4Cl additive. Some IP orientation of RP perovskite can be found at the point of NH_4Cl/PbI_2=1.0, which might be due to the excessive NH_4Cl additive severely suppressed the preformed PDS, making the templated growth of RP perovskite become nonuniform, or alternatively, noncompetitive with homogenous nucleation inside the liquid phase.

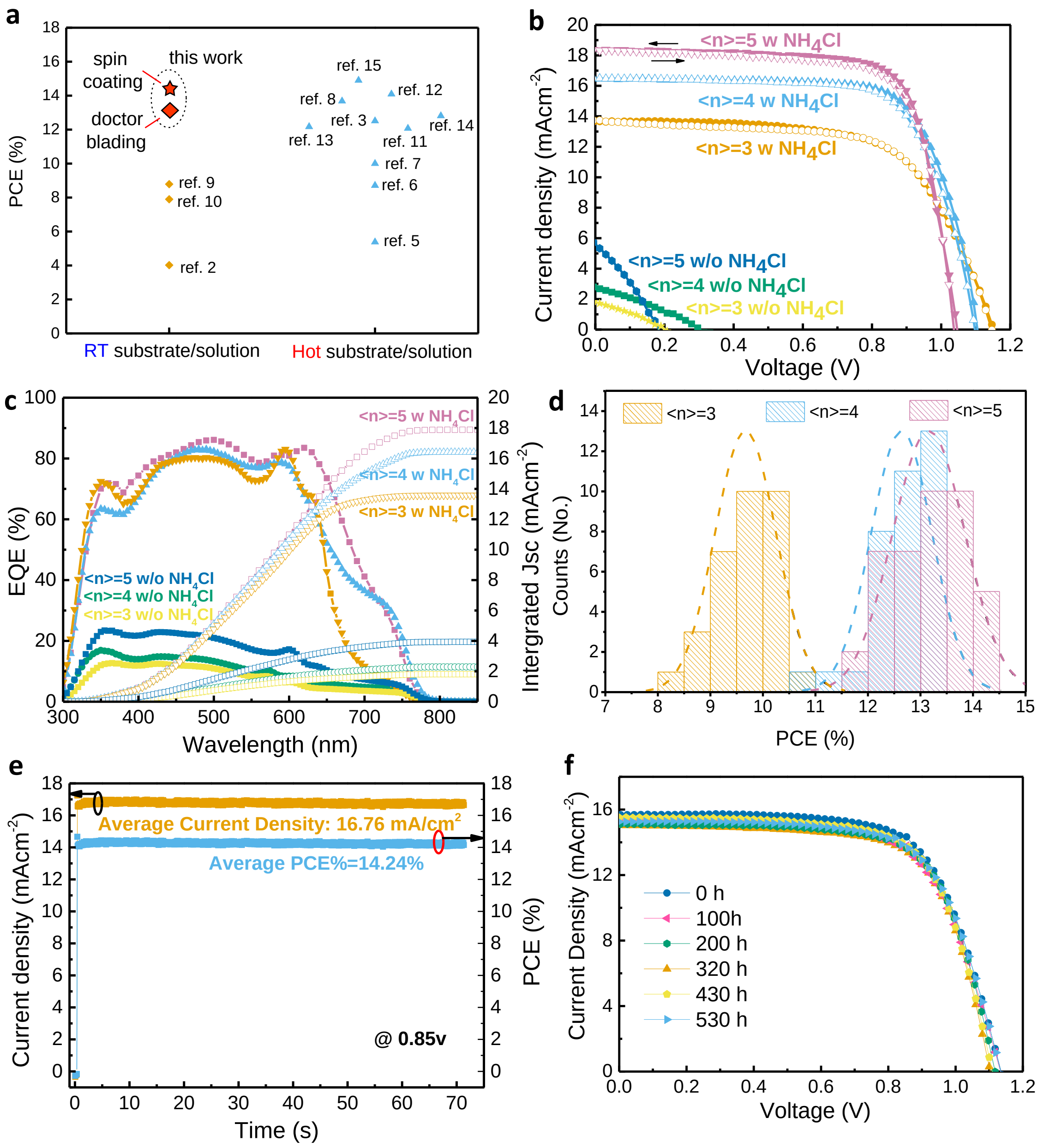

Supplementary Figure 18 | Performance of RP perovskite solar cells (<n>=3-5): (a) Distribution of reported PCEs of BA-based RPPCs (<n>=3-5) fabricated by room-temperature methods or hot-casting method, respectively. (b,c) Current density (*J*)-voltage (*V*) curves (b), external quantum efficiency (EQE) spectra and integrated photocurrents (c) for BA-based RPSCs (<n>=3, 4 and 5) with or without NH_4Cl additives. (d) Statistics of the power conversion efficiency (PCE)

distribution for BA-based RPSCs (<n>=3, 4 and 5) with NH_4Cl additive. (e) Steady state photocurrent and PCE for BA-based RPSCs (<n>= 5) working at a bias of 0.85 V. (f) *J-V* curves of BA-based RPSCs as a function of continuous operation time (aged under one-sun, 100 mW cm^{-2}, with a loading resistance of 1000 ohm), as parameter summarized in Fig. 5c.

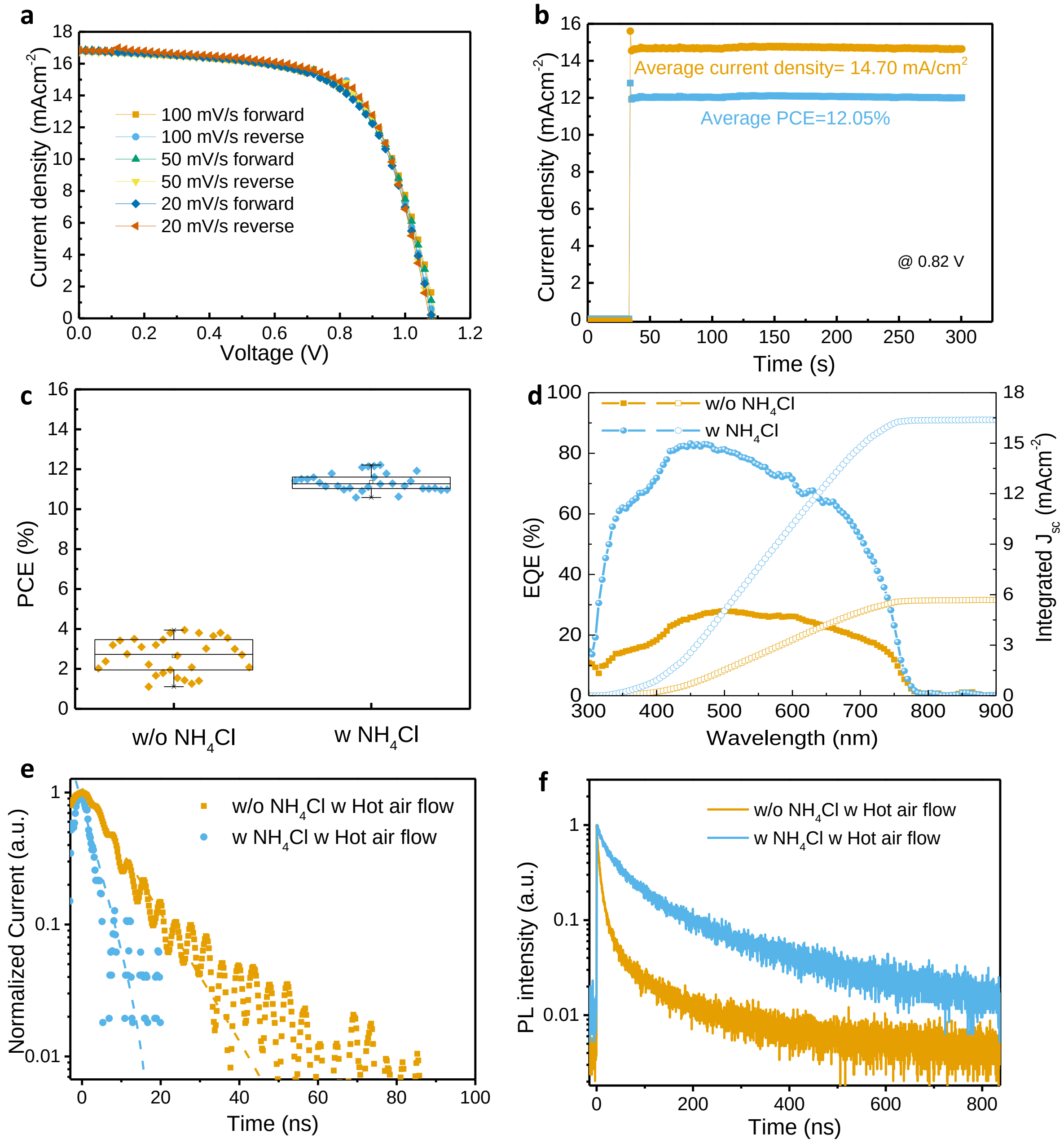

Supplementary Figure 19 | Performance of RPSCs and characterization of charge carriers of RP film by doctor blading. (a) The *J-V* curves for RPSCs (<n>=4) fabricated by doctor blading with additive and hot air flow co-treatment, where changing the scan rates and directions give the same *J-V* curves without hysteresis. (b) Steady state photocurrent and PCE for doctor bladed RPSCs at a

bias of 0.82V. (c-f) Statistics distribution of the PCE (c), EQE spectra and integrated photocurrents (d), Transient photocurrent spectra (e) and Time-resolved photoluminescence spectra (f) of the RPSCs, without additive or with NH_4Cl as additive, fabricated by hot-air-flow assisted doctor blading.

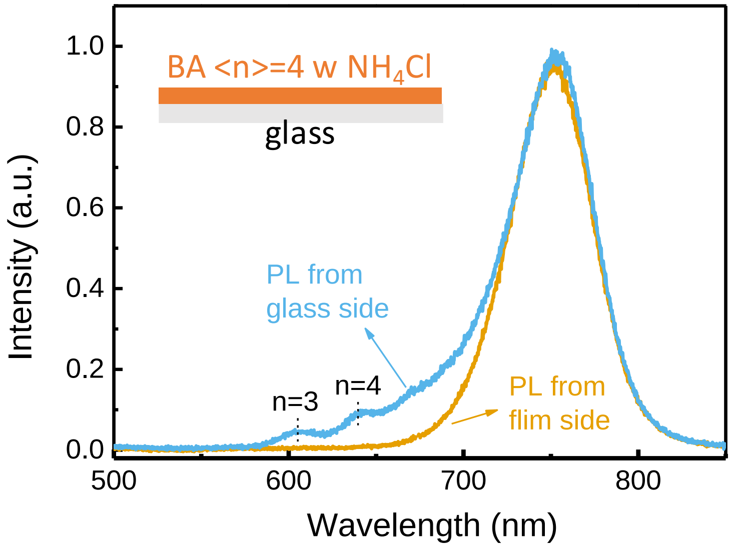

Supplementary Figure 20 | Normalized PL spectra of BA <n>=4 RP perovskite films with NH_4Cl additive under 337 nm laser illumination from perovskite side and glass side. Only PL peak of large-n RP perovskite (~750 nm) was observed when light incident from perovskite film side, PL peaks of small-n RP perovskite (i.e. 605 nm for n=3, 640 nm for n=4) were present when light incident from glass side. The difference on PL spectra indicted that small-n RP perovskite preferred to located at bottom of the film (i.e. near glass side) and large-n RP perovskite preferred to located at the top of the film [4].

Supplementary Tables

Supplementary Table 1. Summary of the performance of reported RP perovskite solar cells (RPSCs) base on $BA_2MA_{n-1}Pb_nI_{3n+1}$ <n>=3-5.

Solar cell structure	Method	V_{OC} [V]	J_{SC} [mA cm^{-2}]	FF [%]	PCE [%]	*Ref*
FTO/c-TiO_2/m-TiO_2/**<n>=3**/ Spiro-OMeTAD/Au	RT SC	0.929	9.42	46	4.02	*2*
FTO/PEDOT:PSS/ **<n>=4**/ PCBM/Al	Hot-casting	1.01	16.76	74.13	12.51	*3*
ITO/C_{60}/ **<n>=4**/ spiro-OMeTAD /Au	Hot-casting & w HI	1.06	11.7	43.38	5.38	*5*
ITO/PEDOT:PSS/**<n>=5**/ PCBM/Al	Hot-casting	1.0	11.44	75.59	8.71	*6*
ITO/PEDOT:PSS/**<n>=5**/ PCBM/Al	Hot-casting & w DMSO	0.986	15.5	65.5	10	*7*
FTO/c-TiO_2/**<n>=4** w 5% Cs^+/ spiro-OMeTAD /Au	Hot-casting & w DMSO	1.08	19.95	63.47	13.68	*8*
ITO/PEDOT:PSS/**<n>=4**/ PCBM/BCP/Ag	RT SC & w NH_4SCN	0.98	14.71	61	8.79	*9*
ITO/PEDOT:PSS/**<n>=4**/ PCBM/Cu	RT SC & DMAc solvent	1.07	11.3	64.8	7.9	*10*
ITO/NiO_x/**<n>=3**/ PCBM: ICBA/BCP/Ag	Hot-casting	1.23	13.61	72.17	12.07	*11*
ITO/PTAA/**<n>=3**/ PCBM/Cr/Au	Hot-casting & w MACl	1.14	14.98	82.5	14.09	*12*
FTO/c-TiO_2/**<n>=4** / spiro-OMeTAD /Au	Hot-casting & w DMSO	1.08	19.45	58.22	12.17	*13*
ITO/PEDOT:PSS/**<n>=4**/ PCBM/BCP/Ag	Hot-casting	0.999	18.12	70.79	12.81	*14*
ITO/PEDOT:PSS/**<n>=4**/ PCBM/PEIE /Ag	RT SC & hot substrate	1.14	18.8	69.5	14.9	*15*
ITO/PEDOT:PSS/**<n>=4**/ PCBM/BCP/Cu	RT SC &NH_4Cl	1.10	16.47	72.74	13.2	*This work*
ITO/PEDOT:PSS/**<n>=5**/ PCBM/BCP/Cu	RT SC &NH_4Cl	1.05	18.35	74.93	14.4	

**c-: compact, m-: mesoporous, RT SC: room temperature spin-coating.*

Supplementary Table 2. Comparison of the photovoltaic parameters of RP solar cells with 0.5 NH_4Cl as additive or without additive achieved in this study. F and R are short for forward scan and reverse scan, respectively.

RP perovskites		Scanning direction	V_{OC} [V]	J_{SC} [mA cm^{-2}]	FF [%]	PCE [%]	J_{EQE} [mA cm^{-2}]
Layer number	Additive						
<n>=3	0.5 NH_4Cl	F	1.15	13.63	64.35	10.1	13.58
<n>=3	0.5 NH_4Cl	R	1.15	13.76	64.03	10.1	-
<n>=4	0.5 NH_4Cl	F	1.10	16.47	72.74	13.2	16.41
<n>=4	0.5 NH_4Cl	R	1.10	16.51	71.18	12.9	-
<n>=5	0.5 NH_4Cl	F	1.05	18.35	74.93	14.4	17.89
<n>=5	0.5 NH_4Cl	R	1.04	18.38	72.96	13.9	-
<n>=3	w/o	F	0.20	1.80	31.70	0.11	1.50
<n>=4	w/o	F	0.31	2.75	30.18	0.25	2.15
<n>=5	w/o	F	0.18	5.61	30.31	0.31	4.01

Supplementary Table 3. The photovoltaic parameters of BA based RP perovskite solar cells (<n>=4) fabricated by doctor blading.

Fabrication condition	V_{OC} [V]	J_{SC} [mA cm^{-2}]	FF [%]	PCE [%]	J_{EQE} [mA cm^{-2}]
w NH_4Cl, w hot air flow	1.06	16.84	68.12	12.2	16.39
w/o NH_4Cl, w hot air flow	1.03	7.21	51.30	3.81	5.69
w NH_4Cl, w/o hot air flow	0.42	2.54	40.31	0.43	-

Supplementary Notes

Supplementary Note 1

Generally, multiple forces can drive the orientation of polygonal crystals or shaped particles in solutions, such as the electrostatic force [16-19], entropic force [20-22], and chemical bonding [23]. Some studies show that planar conjugated molecules [16] or conductive 2D flakes with electron-rich structures (e.g. transition metal dichalcogenides [17,18] or 2D oxide perovskites [19]) favor IP orientations on the conductive substrate because of the inductive electrostatic interaction between electron-rich 2D materials and the substrate besides Van der Waals force. Entropic force is the differential of free energy that is generated when the system tends to increase its entropy, including the formation of low free energy surface, self-assembling under hydrophobic effect [20], shape-sensitive capillary interactions [21], depletion force induced segregation [22] and etc., which rotate polygonal nanocrystals inside the liquid phase [20,22] or at liquid-air interface [20,21]. On the other hand, the chemical bonding plays a significant role in epitaxial growth, making the crystal orientation is largely dependent on the substrate [23]. The nucleation and growth of perovskite crystals in liquid phase could be rather complex since it is ternary (or polynary) system.

Supplementary Note 2

In order to further confirm the preferred precipitation of the PDS phase from BA-based RP perovskite precursor solution, the drying of 20 μL precursor solution at RT was directly observed under an optical microscope (inset of Fig. 2b). It was found that a large amount of PDS always appeared firstly in the precursor solution before red RP perovskite flakes can be formed. When the drying process is slow (e.g. ~10 mins for 20 μL precursor solution), the PDS phase tends to form one dimensional fiber-like structures, which is very similar to the pristine PbI_2-DMF phase formed from PbI_2-DMF solution (inset of Supplementary Figure 4a) and $(MA)_2(DMF)_2Pb_mI_{2m+2}$ (m=3) phases (inset of Supplementary Figure 8a). The presence of DMF molecules is verified by FTIR results, as shown in Supplementary Figure 4a,b.

To determine the composition of the precipitation shown in Figure 3g, we collected the precipitation and carried out XRD measurement. The diffraction peaks of $2\theta=9.06°$ and $2\theta=9.63°$ proves the presence of PbI_2-DMF phase. Besides, intermediate phase of $(MA)_2(DMF)_2Pb_2I_6$ and $(MA)_2(DMF)_2Pb_3I_8$ were also observed, as indicated by the diffraction peaks marked in Supplementary Figure 4c. These $(MA)_2(DMF)_2Pb_mI_{2m+2}$ (m=2,3) phases have been reported to be intermediate steps for the formation of bulk perovskites. As shown in Supplementary Figure 4d, solvated phase like $(MA)_2(DMF)_2Pb_2I_6$ and $(MA)_2(DMF)_2Pb_3I_8$ also possess one dimensional structures and needle-like crystals.

Supplementary Note 3

Soaking the PbI_2-DMF phase into oversaturated RP precursor solution by few seconds leads to the PbI_2-DMF coated with new materials phases (Supplementary Figure 5a), which was taken out and characterized by UV-Vis absorption spectroscope (Fig. 2e), photoluminescence spectroscope (Fig. 2f), and XRD (Supplementary Figure 5b). The red shift of the absorption onset from ~550 nm to ~780 nm and the PL peak around 750 nm suggests the formation of 3D-like corner-sharing PbI_6 octahedra networks with reduced energy bandgap in the corner-sharing PbI_6 octahedra phase. In the XRD study, beyond the $(0\ 1\ \bar{1})$, $(0\ 2\ 0)$ and $(0\ \bar{2}\ 1)$ peaks of PbI_2-DMF located at 2θ=9.06°, 9.63° and 12.28°, respectively, new diffraction peak at 2θ = 14.18° indicates a lattice constant of 6.3 Å, which is assigned to the formed corner-sharing PbI_6 octahedra network on PbI_2-DMF surface. The new diffraction peaks at 2θ=13.53° and 13.68° are recognized as the (0 6 0) peak of RP perovskite with n=2 and the (0 8 0) peak of RP perovskite with n=3, respectively. It was noticed that RP perovskite might also show (1 1 1) peaks around 14.2°. Due to this consideration, the PbI_2-DMF fibers with different soaking time have been measured since the diffraction peaks from the gradually growing RP perovskites will increase with soaking time. As shown in Supplementary Figure 5c, the intensity of the peak at 2θ=14.18° is irrelevant with the increased amount of RP perovskites, i.e. only these peaks from RP perovskites (e.g. 2θ=13.53° and 13.68°) show a dramatic increase with the soaking time. Hence the peak at 2θ=14.18° should be the black colored corner-sharing PbI_6 octahedra phase on PbI_2-DMF surface. The much slower increase of the intensity of 2θ=14.2° peak is probably due to the conversion of PbI_2-DMF into

corner-sharing PbI_6 octahedra phase slowed down when the exposed PbI_2-DMF surface reduced and, on the other hand, the thin corner-sharing PbI_6 octahedra phase was covered by RP perovskite phase, so that the increase of corner-sharing PbI_6 octahedra phase was suppressed. Besides, in our soaking experiment, some $(MA)_2(DMF)_2Pb_3I_8$ phase was also found as indicated by the (1 2 0) and (2 0 0) diffraction peaks located at 2θ=9.53° and 10.26°, respectively, which suggests that the formation of corner-sharing PbI_6 octahedra networks might be (partially) assisted by the formation of $(MA)_2(DMF)_2Pb_3I_8$ as the intermediate phase.

Supplementary Note 4

In our study, we dropped oversaturated RP precursor solution into CB to accelerate the solidifying process of the precursor solution. The construction of result particles was shown in Supplementary Figure 9a, b. The Pb, I, C elements in the region dominated by RP perovskites (region A) and solvate phase (region B) has been confirmed by EDS (Supplementary Figure 9c, d), proving the particles is based on PbI_6^- octahedra. The components in region A and region B were further investigated by spatial resolved infrared spectra in Fig. 2i. While peak at 1370 cm^{-1} could be assigned to BA^+, both BA^+ and MA^+ showed the infrared peaks at 1460 and 1570 cm^{-1}. The micro-flakes at the edge of the particles were determined to contain BA^+ and the bulk part of the particle contained MA^+ and DMF. The presence of 3D-like perovskite was also confirmed by the peak located at ~750 nm in the PL spectra in Supplementary Figure 9e. Thus, the bulk of the particles is identified to 3D-like perovskite coated PDS. Element analysis showed that the I/Pb ratio in region A (~3.3) is larger than that in region B (~3.0). The higher ratio of I/Pb and presence of BA group in region A further confirmed the growth of RP perovskites at the edge of the formed particles.

Supplementary Note 5

In order to study the effect of additives on the crystallization process of precursor solution, we compared the nucleation of precursor solution with NH_4Cl, NH_4I, MACl, MABr and MAI as additives (Fig. 3f, g). By adding antisolvent CB into the precursor to extract DMF rapidly, the precursor solution would be oversaturated until the crystals were precipitated. It was found that the precursor solution without any additives began to precipitate when 540 μl CB was added into 210 μl of BA-based RP perovskite precursor solutions ($\langle n \rangle=4$, Fig. 3g). Meanwhile, all the solutions with AX additives are clear after the injection of CB antisolvent. This experiment shows that the additives can effectively suppress the precipitation of PbI_2-DMF solvated phase.

In our experiment, to quantitatively identify the improved critical concentration of precursor solution for nucleus precipitation (C_n) with additives, the C_n for the nucleation of PbI_2-DMF solvated phase (represented by the concentration of Pb^{2+} ions, see Fig. 3c) in DMF at RT were measured as follows: a spectrum of precursor solution with gradually increased Pb^{2+} ions concentration has been made at 80 °C (by diluting high concentration solution), and then all the solutions were cooled down to RT. Those solutions with Pb^{2+} ions concentration higher than the C_n become cloudy as can be confirmed with Tyndall effect, and on the other hand, all the solutions with Pb^{2+} ions concentration lower than C_n remained clear, from which the C_n can be identified. By using this method, the impacts of the AX additives for the precipitation of PDS in DMF have been figured out as shown in Fig. 3c.

Supplementary Note 6

Supplementary Figure 11 shows the highly reproducible out-of-plane (OP) growth of Ruddlesden–Popper layered perovskites with a variety of AX salts as additives. While the XRD peak at 9° shown up in RP perovskite films without additive indicates the presence of IP orientation of RP perovskites with n=2 (bottom of Supplementary Figure 11a), the absence of XRD peak in the region of 2θ<10° indicates the dominating OP orientation of RP perovskites with excessing AX salts (A=NH_4^+ or MA^+, X= I^-, Br^- or Cl^-). The GIWAXS patterns of the RP perovskites achieved by excessing AX salts (Supplementary Figure 11b) is similar to that of the RP perovskite film with NH_4Cl additive (Fig. 1b), indicating a similar OP dominated crystal orientation caused by excessive AX salts. The concentrated diffraction intensity spots in the GIWAXS further confirms the high crystallinity.

Supplementary Note 7

After the intercalation of MAI, the edge-sharing Pb-I based octahedra chains could rotate to form corner-sharing PbI_6 octahedra chains. The continuous conversion of edge-sharing octahedra chains into corner-sharing PbI_6 octahedra chains with its corners pointing out-of-plane (i.e. with a lattice constant of ~6.3 Å) are geometrically available, i.e. the corner-shared I^- ions between two octahedra can be always kept connected with adjacent Pb^{2+} ions during octahedra rotation (see detail rotation process in Supplementary Movie 1). The reverted process of this kind of conversion has been reported previously by Z. Fan et al.,[24] in which the (1 1 0) orientated 3D perovskite converted into edge-sharing PbI_6 octahedra layers during degradation, agreeing well with our explanation here. On the contrast, the corner-sharing PbI_6 octahedra chains with its edges pointing out-of-plane (i.e. with a lattice constant of ~8.9 Å, as shown in the bottom of Supplementary Figure 12) is unlikely converted from edge-sharing octahedra chains by simple octahedra rotation without breaking considerable Pb-I bonds. Due to this reason, the orientation of RP crystal grown on PDS surface is dominated by the corner-sharing PbI_6 octahedra chains with its corners pointing out-of-plane, which explains the (n 0 0) or (0 0 n) diffraction peaks of RP perovskite is barely seen even if the RP perovskite crystals have OP orientation.[2,3,9]

Supplementary Note 8

The effect of NH_4Cl on the orientation of PEA-based RP type layered perovskite and PPD based DJ type layered perovskite have been studied by GIWAXS and XRD characterizations. As shown in Supplementary Figure 13a, b, in the sample with NH_4Cl as additive, the concentrated diffraction intensity spots and disappearing of diffraction along q_z axis in the range of 0~10 nm^{-1} in the GIWAXS indicated that the OP orientation was also achieved for PEA-based RP perovskite, which was prepared by the same method as that for BA-based RP perovskite. The OP orientation of PEA-based RP perovskite was also confirmed by XRD in Supplementary Figure 13c, where the (1 1 1) and (2 0 2) peaks become dominating after adding NH_4Cl and the (0 6 0) peaks at 2θ=11.11° related to IP orientation disappeared. Similarly, the disappear of (0 8 0) peaks at 2θ=11.39° and the increasing of (1 1 1) and (2 0 2) peaks indicate the dominated OP orientation in PPD based DJ perovskite achieved by adding NH_4Cl.

Supplementary Note 9

Supplementary Table 1 and Supplementary Figure 18a summarized the reported PCEs of RPSCs based on butylamine (BA) as long-chain organic cation. We highlight that our RPSCs were prepared with both solution and substrate at room temperature and the achieved PCEs are 13.2% and 14.4% for <n>=4 and 5, respectively. While the open circuit voltage (V_{OC}) for BA-based RP perovskite solar cells with <n>=3, 4 and 5 dropped from 1.15 to 1.10 and 1.05 V, respectively, the short circuit current density (J_{SC}) increased from 13.63 to 16.47 and 18.35 mA cm^{-2} and the fill factor (FF) increased from 64% to 73% and 75%, respectively (details in Supplementary Table 2 and Supplementary Figure 18b). The increase of J_{SC} and FF may be due to the improved absorption and better charge transporting efficiency of BA <n>=4 and 5 films, the decreasing of V_{OC} may be due to the reduced bandgap of the BA <n>=4 and 5. The integrated J_{SC} from EQE spectra (J_{EQE}) matched well with J_{SC} from J-V curves with a small variation of about ~2% (Supplementary Figure 18c). The statistics of the PCE distribution for RP perovskite solar cells with <n>=3, 4, and 5, are shown in Supplementary Figure 18d, which demonstrates the reliability of the PCEs achieved in RPSCs with NH_4Cl as additives. The steady state photocurrent and PCE for RPSCs with <n>=5 measured at 0.85V was shown in Supplementary Figure 18e, confirming the device performance parameters extracted from the *J-V* curves and demonstrating the ignorable photocurrent hysteresis in our devices. Supplementary Figure 18f indicated good stability of our RPSCs working at maximum power point at one-sun illumination.

Supplementary Note 10

In Supplementary Figure 19a, no obvious hysteresis effect was observed for our RPSCs under different voltage scan rate. The statistic distribution of obtained PCE in Supplementary Figure 19c indicates good device repeatability for the doctor bladed RPSCs. The integrated *J*sc from EQE spectra (16.39 mA cm^{-2}) agreed with the *J*sc from *J-V* curves (16.84 mA cm^{-2}) with a small error of less than 3% (Supplementary Figure 19d). The charge carrier mobility of charge carriers for RPSCs (<n>=4) with and without NH_4Cl was measured by transient photocurrent (TPC) method. The transition time (t) for photogenerated charge carriers to be swept out of the RP perovskite layer (with d=270 nm) follows the equation of $\mu = d^2\ t^{-1}\ V_{Bi}^{-1}$, where μ is the carrier mobility and V_{Bi} is the build-in voltage. The shortened transition time from 9.1 ns to 2.9 ns by using NH_4Cl additive in the doctor bladed RP perovskite film suggested an improved mobility from ~0.076 $cm^2\ V^{-1}\ s^{-1}$ to 0.24 $cm^2\ V^{-1}\ s^{-1}$ (Supplementary Figure 19e). On the other hand, the PL lifetime were 48 and 6 ns for RP perovskite films (<n>=4) with and without NH_4Cl, respectively. This increased mobility and PL lifetime by using NH_4Cl additives benefits from the improved crystallinity and dominated OP orientation.

Supplementary References

1. Stoumpos, C. C. et al. Ruddlesden-Popper Hybrid Lead Iodide Perovskite 2D Homologous Semiconductors. *Chem Mater* **28,** 2852-2867, (2016).
2. Cao, D. H., Stoumpos, C. C., Farha, O. K., Hupp, J. T. & Kanatzidis, M. G. 2D Homologous Perovskites as Light-Absorbing Materials for Solar Cell Applications. *J. Am. Chem. Soc.* **137**, 7843-7850, (2015).
3. Tsai, H. *et al.* High-efficiency two-dimensional Ruddlesden–Popper perovskite solar cells. *Nature* **536**, 312-316, (2016).
4. Liu, J., Leng, J., Wu, K., Zhang, J. & Jin, S. Observation of Internal Photoinduced Electron and Hole Separation in Hybrid Two-Dimentional Perovskite Films. *J. Am. Chem. Soc.* **139,** 1432-1435, (2017).
5. Chen, Y. N. *et al.* Tailoring Organic Cation of 2D Air-Stable Organometal Halide Perovskites for Highly Efficient Planar Solar Cells. *Adv. Energy Mater.* **7**, 1700162,(2017).
6. Stoumpos, C. C. *et al.* High Members of the 2D Ruddlesden-Popper Halide Perovskites: Synthesis, Optical Properties, and Solar Cells of (CH 3 (CH 2) 3 NH 3) 2 (CH 3 NH 3) 4 Pb 5 I 16. *Chem.* **2**, 427-440, (2017).
7. Soe, C. M. M. *et al.* Understanding Film Formation Morphology and Orientation in High Member 2D Ruddlesden-Popper Perovskites for High-Efficiency Solar Cells. *Adv. Energy Mater.* **8**, 1700979, (2018).
8. Zhang, X. *et al.* Stable high efficiency two-dimensional perovskite solar cells via cesium

doping. *Energ. Environ. Sci.* **10**, 2095-2102, (2017).

9. Zhang, X. *et al.* Vertically Oriented 2D Layered Perovskite Solar Cells with Enhanced Efficiency and Good Stability. *Small* **13**, 1700611, (2017).

10. Chen, A. Z. et al. Origin of vertical orientation in two-dimensional metal halide perovskites and its effect on photovoltaic performance. *Nat. Commun.* **9,** 1336, (2018).

11. Chen, J. *et al.* Interfacial engineering enables high efficiency with a high open-circuit voltage above 1.23 V in 2D perovskite solar cells. *J. Mater. Chem. A* **6**, 18010-18017, (2018).

12. Yang, R. *et al.* Oriented Quasi-2D Perovskites for High Performance Optoelectronic Devices. *Adv. Mater.* **30**, e1804771, (2018).

13. Zhang, X. *et al.* Phase Transition Control for High Performance Ruddlesden-Popper Perovskite Solar Cells. *Adv. Mater.* **30**, e1707166, (2018).

14. Zhou, N. *et al.* Exploration of Crystallization Kinetics in Quasi Two-Dimensional Perovskite and High Performance Solar Cells. *J. Am. Chem. Soc.* **140**, 459-465, (2018).

15. Zuo, C. *et al.* Self-Assembled 2D Perovskite Layers for Efficient Printable Solar Cells. *Adv. Energy Mater.* **0**, 1803258, (2018).

16. Wang, S. T. et al. Interface electronic structure and morphology of 2,7-dioctyl[1]benzothieno[3,2-b]benzothiophene (C8-BTBT) on Au film. *Appl. Surf. Sci.* **416,** 696-703, (2017).

17. Novoselov, K. S., Mishchenko, A., Carvalho, A. & Castro Neto, A. H. 2D materials and van der Waals heterostructures. *Science* **353,** (2016).

18. Zeng, M., Xiao, Y., Liu, J., Yang, K. & Fu, L. Exploring Two-Dimensional Materials toward the Next-Generation Circuits: From Monomer Design to Assembly Control. *Chem. Rev.* **118,** 6236-6296, (2018).

19. Ebina, Y., Sasaki, T., Harada, M. & Watanabe, M. Restacked perovskite nanosheets and their Pt-loaded materials as photocatalysts. *Chem. Mater.* **14,** 4390-4395, (2002).

20. Rycenga, M., McLellan, J. M. & Xia, Y. N. Controlling the assembly of silver nanocubes through selective functionalization of their faces. *Adv. Mater.* **20,** 2416-+, (2008).

21. Yunker, P. J., Still, T., Lohr, M. A. & Yodh, A. G. Suppression of the coffee-ring effect by shape-dependent capillary interactions. *Nature* **476,** 308-311, (2011).

22. Zhao, K. & Mason, T. G. Directing colloidal self-assembly through roughness-controlled depletion attractions. *Phys. Rev. Lett.* **99,** 268301, (2007).

23. Vesselinov, M. I. Crystal growth for beginners: fundamentals of nucleation, crystal growth and epitaxy. (World scientific, 2016).

24. Fan, Z. et al. Layer-by-Layer Degradation of Methylammonium Lead Tri-iodide Perovskite Microplates. *Joule* **1,** 548-562, (2017).
