# Supplementary material for: Templated growth of oriented layered hybrid perovskites on 3D-like perovskites
Source: Nat Commun. 2020 Jan 29;11:582. doi: 10.1038/s41467-019-13856-1 (PMC6989653; doi:10.1038/s41467-019-13856-1)
Supplement: Supplementary file 3 — Description of Additional Supplementary Files [file 41467_2019_13856_MOESM3_ESM.pdf]

**Description of Additional Supplementary Files**

File Name: Supplementary Movie 1
Description: The illustration of the conversion of edgesharing $PbI_6$ octahedra chains in solvated phase to the corner-sharing PbI6 octahedra chains in 3D-like perovskite phase by MAI intercalation.
